# Analysis of the Salivary Gland Transcriptome of *Frankliniella occidentalis*

**DOI:** 10.1371/journal.pone.0094447

**Published:** 2014-04-15

**Authors:** Candice A. Stafford-Banks, Dorith Rotenberg, Brian R. Johnson, Anna E. Whitfield, Diane E. Ullman

**Affiliations:** 1 Department of Plant Pathology, University of California Davis, Davis, California, United States of America; 2 Department of Plant Pathology, Kansas State University, Manhattan, Kansas, United States of America; 3 Department of Entomology, University of California, Davis Davis, California, United States of America; Zhejiang University, China

## Abstract

Saliva is known to play a crucial role in insect feeding behavior and virus transmission. Currently, little is known about the salivary glands and saliva of thrips, despite the fact that *Frankliniella occidentalis* (Pergande) (the western flower thrips) is a serious pest due to its destructive feeding, wide host range, and transmission of tospoviruses. As a first step towards characterizing thrips salivary gland functions, we sequenced the transcriptome of the primary salivary glands of *F. occidentalis* using short read sequencing (Illumina) technology. A *de novo*-assembled transcriptome revealed 31,392 high quality contigs with an average size of 605 bp. A total of 12,166 contigs had significant BLASTx or tBLASTx hits (E≤1.0E^−6^) to known proteins, whereas a high percentage (61.24%) of contigs had no apparent protein or nucleotide hits. Comparison of the *F. occidentalis* salivary gland transcriptome (sialotranscriptome) against a published *F. occidentalis* full body transcriptome assembled from Roche-454 reads revealed several contigs with putative annotations associated with salivary gland functions. KEGG pathway analysis of the sialotranscriptome revealed that the majority (18 out of the top 20 predicted KEGG pathways) of the salivary gland contig sequences match proteins involved in metabolism. We identified several genes likely to be involved in detoxification and inhibition of plant defense responses including aldehyde dehydrogenase, metalloprotease, glucose oxidase, glucose dehydrogenase, and regucalcin. We also identified several genes that may play a role in the extra-oral digestion of plant structural tissues including β-glucosidase and pectin lyase; and the extra-oral digestion of sugars, including α-amylase, maltase, sucrase, and α-glucosidase. This is the first analysis of a sialotranscriptome for any Thysanopteran species and it provides a foundational tool to further our understanding of how thrips interact with their plant hosts and the viruses they transmit.

## Introduction

A diverse range of salivary components are known to play a crucial role in the successful feeding of phytophagous insects. Regardless of the specific feeding strategy used, insect saliva is secreted for the suppression and detoxification of plant defense responses and the extra-oral digestion of plant tissues. Caterpillars, which have chewing mouthparts, secrete glucose oxidase (GOX) to suppress wound-induced plant defenses [1], [2]. Saliva secreted by aphids, which have piercing/sucking mouthparts and specialize on phloem sap, counteracts calcium-triggered occlusion of phloem sieve tubes [3], [4]. Polygalacturonases secreted by Lygus bugs, which have piercing/sucking mouthparts and utilize a cell rupture feeding strategy, enzymatically digest plant tissues for subsequent ingestion [5]. The application of an individual salivary component to a host plant is sufficient to mimic insect feeding damage [6] and when insects are unable to produce certain salivary components, survival dramatically decreases [1], [7]. Knowledge of salivary secretions is crucial to understanding how insects interact with their host plants and a deep understanding of insect-plant interactions will facilitate the development of better pest control strategies. Despite their importance as pests, there is currently little known about the secretion and composition of thrips saliva.

*Frankliniella occidentalis* (Pergande) (the western flower thrips) is an important agronomic pest due to economic losses on a wide range of host plants caused by direct feeding damage and tospovirus transmission, which occurs during salivation. Thrips are the only insects that vector *Tospoviruses*, the plant-infecting genus in the family *Bunyaviridae*. *F. occidentalis* is the most efficient tospovirus vector, transmitting five of the eight officially-approved species [8]. In many locations, *F. occidentalis* is the primary vector of *Tomato spotted wilt virus* (TSWV), type member of the *Tospovirus* genus. Male thrips are more efficient vectors than female thrips and it has been hypothesized that a more robust virus infection in male thrips and sexually dimorphic feeding behaviors may be responsible for increased transmission efficiency in males [9]–[12]. Thrips have piercing/sucking mouthparts and a unique punch and suck feeding strategy whereby they feed on individual epidermal and mesophyll cells by piercing the cells with their stylets, then sucking out the cell contents [12], [13]. Thrips also engage in sustained ingestion from an unknown plant tissue, perhaps xylem sap or contents of previously ruptured cells [12]–[14]. Evidence supports the hypothesis that thrips engage in cell rupture feeding, because prolonged thrips feeding results in extensive pockets of cell damage, including cell wall destruction [13], [15]. During cell rupture feeding, thrips likely macerate tissues with their stylets and secrete saliva to extra-orally digest plant tissue, and then subsequently feed on the leaking cell contents.

Salivation has been directly observed before and during some specific types of thrips feeding behaviors but it likely plays a role in the initiation of all types of probing behaviors [12]. Like homopterous insects, thrips produce watery and gelling salivary secretions but because they produce such tiny amounts of saliva it is very difficult to collect. Thrips watery saliva diffuses throughout plant cells and likely plays a role in lubrication of the mouthparts, extra-oral digestion of cell contents and structural components, and prevention of plant defense responses [16], [17]. Gelling saliva does not diffuse but localizes to the sites it is produced in, likely playing a role in the prevention of sap leakage, adhesion of the mouthcone to the leaf surface, and sealing of the stylets into a cell under turgor pressure [15]–[19].

Thysanopterans have two pairs of salivary glands, the ovoidal primary glands and the tubular secondary glands [20]–[25]. The primary salivary glands are composed of large, loosely aggregated, binucleate cells and the secondary tubular salivary glands are formed by a flattened monolayer of cells. It is unclear which type of saliva is produced in each of the salivary gland cells but the extremely different morphology of each gland suggests that they may produce salivary compounds with different functions. Viral proteins have been observed in both types of salivary glands [23], [26] indicating that both primary and tubular salivary glands may play an important role in virus transmission. Although thrips salivary glands have been described, little is known about the biological, chemical, and molecular features of thrips salivary glands and saliva. Virtually nothing is known about the mechanisms of saliva production and secretion, the composition of thrips saliva, or the salivary proteins involved in plant-insect and virus-vector interactions.

The vast majority of salivary gland transcriptome studies, known as sialomes or sialotranscriptomes, are from blood feeding insects, including: *Anopheles gambiae* Giles [27], [28], *Aedes aegy*pti (L.) [29], *Aedes albopictus* (Skuse) [30], *Simulium vittatum* Zetterstedt [31], *Triatoma infestans* (Klug) [32] *Glossina morsitans morsitans* Westwood [33] and *Ixodes scapularis* Say [34]. However, the advent of multiple next generation sequencing platforms has enabled genomic studies of non-model organisms including phytophagous insects such as whiteflies [35], and leafhoppers [36]. By far the best studied group of piercing-sucking phytophagous insects are the aphids, whose salivary glands and saliva have been well characterized through genomic and proteomic studies [37]–[41].

Recently genomic and proteomic tools have been developed for thrips, including a collection of assembled and annotated sequences, constructed from two different whole body thrips transcriptomes [42], [43]. In this paper we present the first sialotranscriptome for a member of the order Thysanoptera. The thrips sialotranscriptome we describe provides a foundational view of thrips salivary components essential to understanding how thrips feed and interact with tospoviruses.

## Materials and Methods

### Thrips samples and salivary gland collection

*F. occidentalis* and TSWV isolate MT2 were originally collected in Hawaii. Thrips populations were reared on green bean pods (*Phaseolus vulgaris* L.) as previously described [44]. TSWV was maintained in *Emilia sonchifolia* (L.) plants and passaged by thrips transmission as previously described [44]. Thrips were age regulated by isolating individuals at the second pupal stage then removing them upon adult eclosion to guarantee that all thrips differed in age by no more than 24 hours. Salivary glands were dissected for three biological replicates of four treatment groups: 1) TSWV-infected males, 2) uninfected males, 3) TSWV-infected females, and 4) uninfected females. TSWV infected and uninfected thrips were obtained as previously described [44]. To ensure the infection status of all treatments a subsample of 10 thrips was taken from the age regulated population and their infection status verified by a transmission assay as previously described [10]. Thrips salivary glands were dissected in cold 60% molecular grade, RNase free, ethanol on glass microscope slides. All tools were sterilized in a 10% bleach solution and treated with RNase zap (Life Technologies, Ambion, USA) between dissections to reduce cross contamination between samples.

### RNA isolation and Illumina sequencing

For RNA extraction, multiple samples were pooled because each salivary gland only consists of about 8 cells [24]. Roughly 100 female primary salivary glands, and 200 male primary salivary glands were dissected and pooled together for each sample. After dissection salivary glands were immediately placed into 50 µl of cold extraction buffer. Total RNA was isolated using the PicoPure RNA Isolation Kit (Life Technologies, USA) according to the manufacturer's protocol. RNA quantity and quality was assessed by Nanodrop ND-1000 (Thermo Scientific, DE, USA) UV/Vis spectroscopy, and DNA bioanalyzer 2100 (Agilent, CA, USA) microfluidics, respectively.

Total RNA samples were submitted to the University of California, Davis Genome Center, DNA Technologies Core for cDNA library construction and Illumina sequencing. Due to the low concentration of total RNA in the samples (around 200 ng) cDNA was made directly from total RNA using random hexamers. Double-stranded (ds) cDNA overhang fragments were end-repaired by incubation in the presence of T4 DNA polymerase and Klenow polymerase. Polished fragments were phosphorylated by T4 PNK, followed by the addition of a single ‘A’ base to the 3′ end of the blunt-ended phosphorylated fragments for ligation to proprietary adapter oligonucleotides for Illumina multiplexed paired-read sequencing. To remove abundant transcripts such as rRNA while preserving molecules derived from less abundant transcripts, the library was normalized by treating with duplex-specific thermostable nuclease (DSN), (Evrogen) for 25 minute at 68°C [45]. The DSN treated RNA-seq library was purified with SPRI beads and subjected to a final PCR amplification (10 cycles). The 12 amplified libraries were quantitatively and qualitatively assessed by Nanodrop ND-1000 (Thermo Scientific, DE, USA) UV/Vis spectroscopy, DNA bioanalyzer 2100 (Agilent, CA, USA) microfluidics, and realtime quantitative PCR (Kapa Biosystem, USA) to determine high quality molecules for pooling of libraries at equimolar concentration and the subsequent sequencing on Illumina HiSeq2500 for paired read of 100 or 105 bases following manufacturer's manual. Sample-specific tags were added to the RNAseq libraries to differentiate the sample reads after multiplexing. One sample from each treatment group was pooled together for multiplex sequencing on an individual lane for a total of 3 lanes and 12 samples. The Illumina reads were deposited into the NCBI Short Read Archive (SRA) under study accession number SRP036079. Assembled sequences which conformed to NCBI standards were deposited into the Transcriptome Shotgun Assembly (TSA) at DDBJ/EMBL/GenBank under the accession GAXD00000000. The version described in this paper is the first version, GAXD01000000.

### Bioinformatics data analysis

Transcriptome assembly and annotation was performed by the Bioinformatics Core at the University of California, Davis Genome Center. Initial quality control was performed on the raw sequences using R Bioconductor software package (version 2.9, DESeq volume 1.6.0, qrqc developmental version) to assess overall read quality, Scythe (developmental version) to remove any 3′ adapter contamination, Sickle (version 1.0) to trim low-quality sequences, and Sabre (1.0) to de-multiplex and remove barcodes. Prior to assembly, all reads were aligned to a concatenated fasta consisting of the pea aphid genome [46], the TSWV genome, the grape genome, and all *F. occidentalis* nucleotide sequences from NCBI. All alignments were done with bwa (version 0.5.9). The pea aphid genome was not sufficiently close to the *F. occidentalis* reads to be used as a reference genome (<15% of FO reads aligned to pea aphid with default parameters); therefore the *F. occidentalis* sialotranscriptome was assembled *de novo*. Subassemblies were made for each lane of Illumina sequencing then combined for the full assembly. Contigs were assembled *de novo* using Velvet (version 1.1.05)/Oases (version 0.1.22) at four k-mers (21, 37, 55, and 71). Contigs less than 200 bp and those with long stretches of Ns were removed. The subassemblies with kmers 21, 37, and 55 were run with the trimmed reads as input. The transcript contigs from these assemblies were then trimmed to remove Ns and these trimmed transcript contigs, along with the original trimmed reads, were used as input to the velvet/oases assembly with kmer 71. The output contigs from the kmer 71 assembly were further processed to remove excess Ns, and were then clustered using tgicl/CAP3 to produce the final transcriptome assembly.

After assembly all reads were aligned to the transcriptome to determine the read contributions of each treatment. At this step, contamination from rRNA was discovered to be very abundant (92.1%). There was adequate coverage to gain useful information about this novel transcriptome; however, we were unable to quantify gene expression as initially hoped. The reads were independently aligned to the *F. occidentalis* full body transcriptome to aid in gene annotation [43]. Statistical analyses between the two transcriptomes were not performed due to the different methods and insect materials used to generate the transcriptomes, nor were hybrid assemblies generated with the two transcriptomes. Reciprocal blasts were run between the *F. occidentalis* sialotranscriptome and the full body transcriptome [43] to generate a final annotation spreadsheet with consolidated results from all blasts, annotation, and comparisons between the assemblies. Annotations were performed using a BLASTx algorithm against swissprot and nr protein sequence databases, and tBLASTx algorithm against the NCBI nr nucletotide database to determine protein coding as well as non-coding sequences with a cutoff value of E-value≤1.0E^−6^ [47]. Functional annotation by GO term was analyzed by Blast2GO with a cutoff value of E-value≤1.0E^−6^ [48].

## Results and Discussion

### Illumina Sequencing and Read Assembly

**Figure 1:**
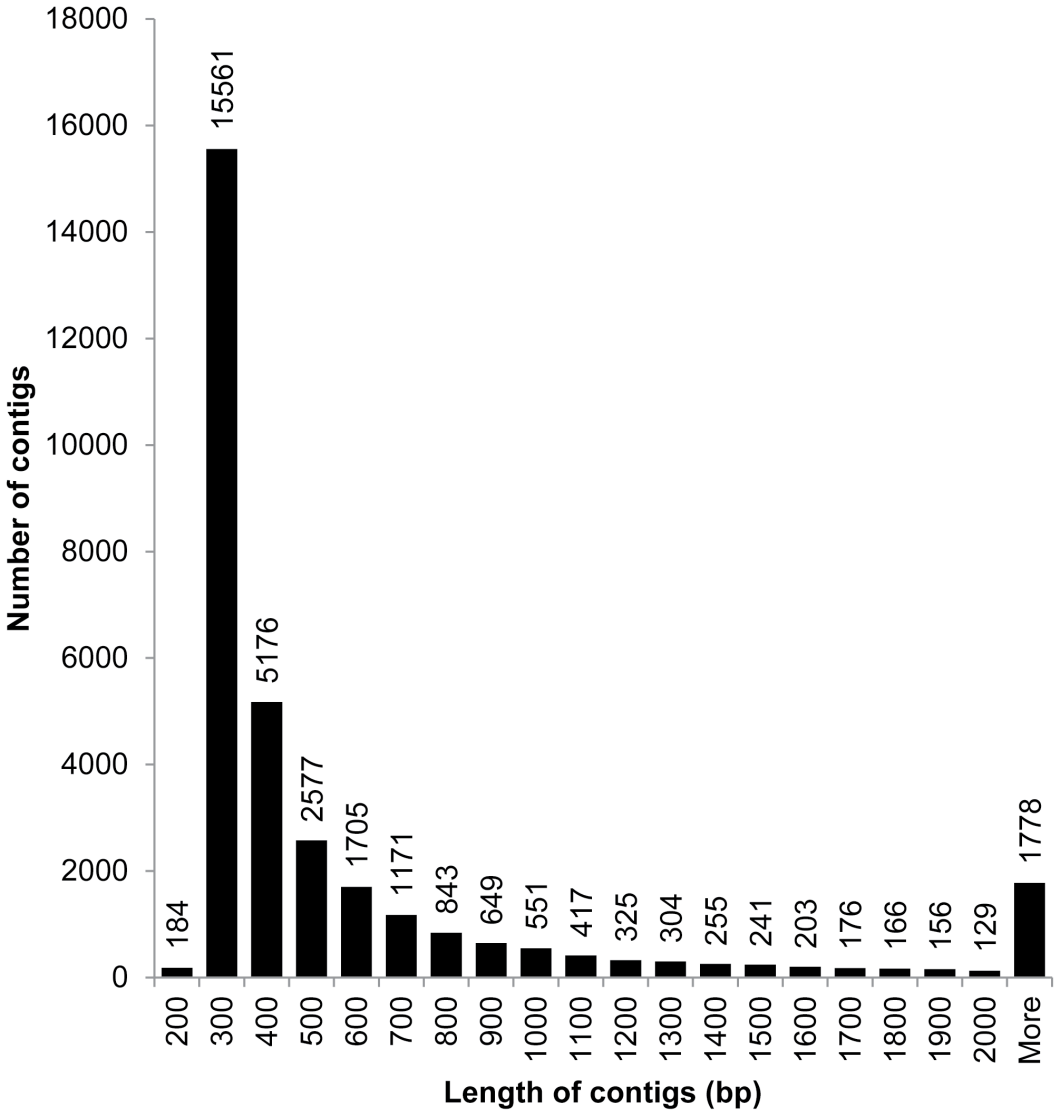
Size distribution of *Frankliniella occidentalis* sialotranscriptome contigs.

The Illumina sequencing of infected and uninfected male and female thrips salivary glands produced in total approximately 405 million high quality 100 or 105 bp paired end reads (Table 1). These reads assembled into 31,392 high quality contigs, including contigs that mapped to viral, bacterial, and fungal genes (Table S1). Contigs ranged in size from 200-14,469 bp, with an average size of 605 bp (Figure 1). Each treatment group contributed reads to the overall assembly (Table 2). Five contigs mapped to the three genome segments of *Tomato spotted wilt virus*, with almost complete coverage of the full length of each genome segment (Table 3). This is the first report of TSWV sequence information from the salivary glands of thrips. Over 90% of reads from all treatment groups (92% of total reads) aligned to ribosomal RNA of *F. occidentals* and related Thysanoptera (Table 2). A small number of reads aligned to TSWV RNA from all treatment groups and was most likely due to contamination with virus particles during dissection or sample processing. Infected treatment groups had substantially more viral RNA than the uninfected treatment groups, with the exception of infected female and male rep 3 which had very low viral titers and transmission rates (10% transmission rate) compared to the other infected groups (70–100% transmission rates) (Table 2).

**Table 1:** Summary of the *Frankliniella occidentalis* sialotranscriptome.

Total number of reads	430,255,071
Total base pairs	34,428,987,775 bp
Total number of high quality reads	405,046,915
Read length	100 or 105 bp (paired end)
Reads aligned to rRNA	373,087,891 (92.1%)
Reads aligned to TSWV	13,205 (<1%)
Reads used for sialotranscriptome assembly	31,945,819 (7.9%)
Total base pairs in assembly	19,729,628 bp
Total number of contigs *	31,392
Mean length of contigs	605 bp

*After the removal of all rRNA sequences.

**Table 2:** Sequence contributions and read alignments from treatment groups that contributed to the *Frankliniella occidentalis* sialotranscriptome.

Treatment*	Input sequences	Assembly reads aligned	rRNA reads aligned	TSWV reads aligned	Total reads aligned
**Total**	430,255,071	31,945,819	373,087,891	13,205	405,046,915
**UF-1**	41,716,701	3,795,951	34,695,943	3	38,491,897
**UF-2**	37,831,399	2,462,606	33,585,955	4	36,048,565
**UF-3**	17,319,603	1,397,098	15,056,023	5	16,453,126
**IF-1**	40,557,520	2,946,585	35,160,876	2,929	38,110,390
**IF-2**	37,185,747	2,888,932	32,111,260	1,348	35,001,540
**IF-3**	40,461,648	2,905,468	35,308,091	8	38,213,567
**UM-1**	38,789,211	2,619,930	34,111,510	16	36,731,456
**UM-2**	31,478,854	2,167,344	27,541,049	11	29,708,404
**UM-3**	31,436,998	2,287,260	27,478,541	1	29,765,802
**IM-1**	33,964,265	2,481,873	29,289,430	2,334	31,773,637
**IM-2**	41,686,916	3,041,355	36,187,019	6,373	39,234,747
**IM-3**	37,826,209	2,951,417	32,562,194	173	35,513,784

* UF- uninfected female, IF- infected female, UM- uninfected male, IM- infected male.

**Table 3:** Representation of all three genome segments of *Tomato spotted wilt virus* in the *Frankliniella occidentalis* sialotranscriptome.

Contig ID	Virus genome segment	Contig length	% coverage of genome segment	E-value	Sequence Identity (%)*
THRIPS_11_08_2011_22063	**L** 1	7349	82	0	97
THRIPS_11_08_2011_22064	**L**	8921	99	0	97
THRIPS_11_08_2011_00971	**M** 2	4840	99	0	99
THRIPS_11_08_2011_01563	**S** 3	6127	93	0	98
THRIPS_11_08_2011_26450	**S**	2806	88	0	99

*Sequence identity determined by BLAST N comparison of contigs with TSWV sequences in GenBank (L segment: KC261971.1, M segment: AY744491.1, and S segment: AY744477.1).

1 L:Large RNA segment that codes for virus RNA-dependent RNA-polymerase protein.

2 M: Medium RNA segment that codes for virus Glycoprotein (G_N_ and G_C_) polyprotein precursor and NSm proteins.

3 S: Small RNA segment that codes for virus nucleocapsid and NSs proteins.

### Transcript Annotation

**Figure 2:**
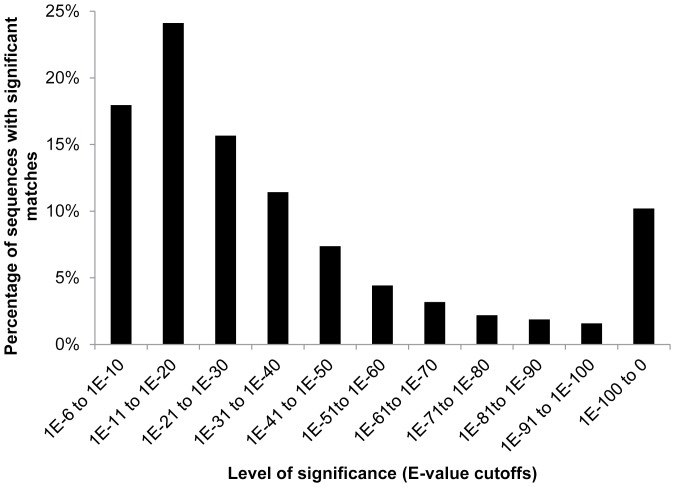
Distribution of E-value of BLAST hits for each contig in the *Frankliniella occidentalis* sialotranscriptome with a cut-off E-value of 1.0E^−6^.

**Figure 3:**
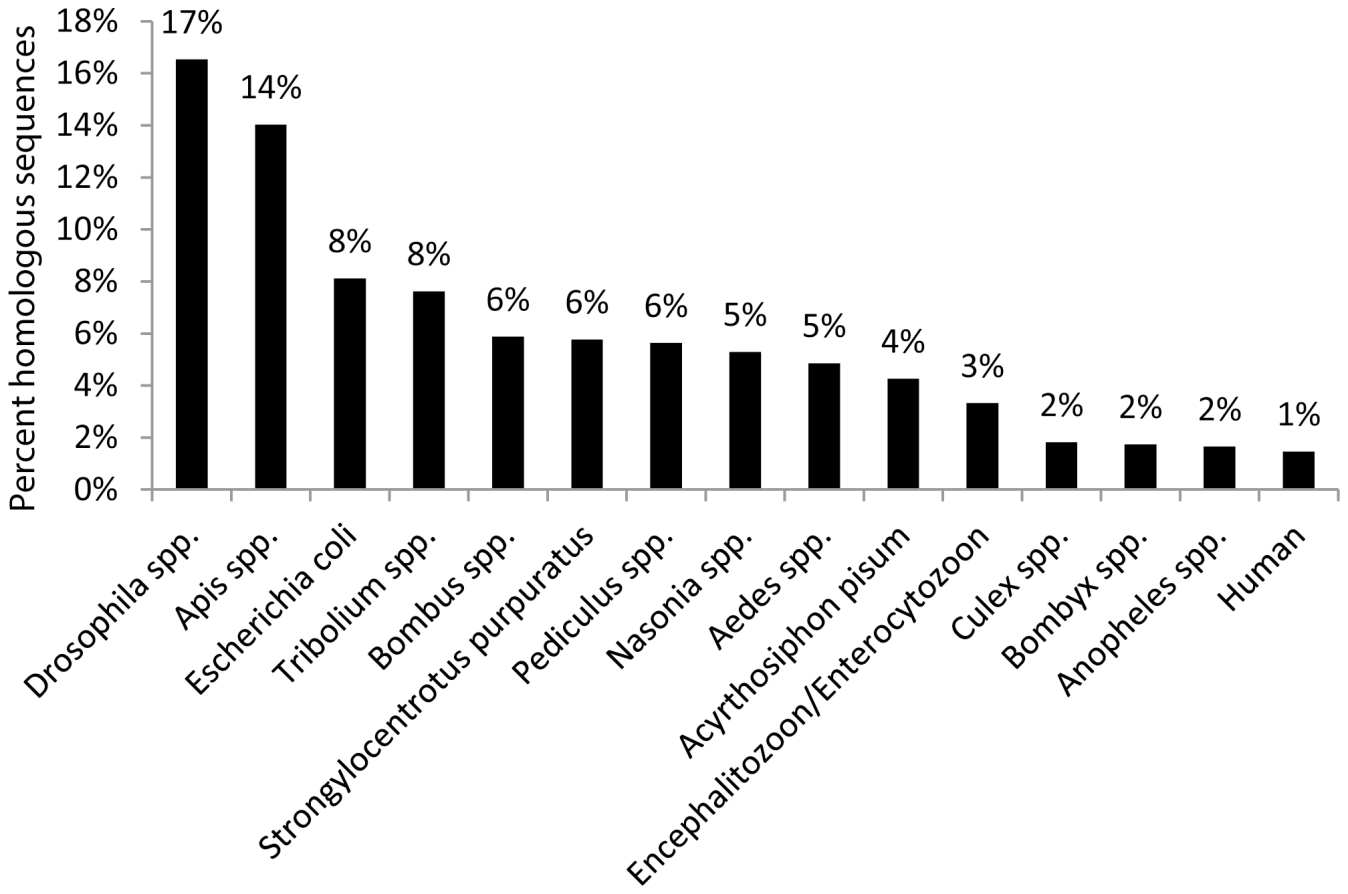
Top 15 species distribution of homologous sequences with an E-value cut-off of 1.0E^−6^ in the *Frankliniella occidentalis* sialotranscriptome.

Two parallel annotations of the 31,392 contigs were performed. In the first annotation BLASTx was run against swissprot and nr protein sequence databases, and InterProScan was performed to derive annotation from putative protein motifs. Subsequently, BLAST2GO mapping and annotation steps were run to assign function and enzyme codes. In the second annotation tBLASTx was run against the NCBI nr nucleotide database to determine protein coding as well as non-coding sequences. A total of 12,166 contigs had significant BLAST hits (E-value≤1.0E^−6^) (Table S2). Of these contigs, 24% had highly significant matches to known proteins (E-value≤1.0E^−50^) (Figure 2). The highest percentage of top blast hits came from *Drosophila* species (17%) and Apis species (14%) (Figure 3). Interestingly *Acyrthosiphon pisum* (Harris) and *Tribolium castaneum* (Herbst) had a much lower percentage of sequence similarity (4% and 8% respectively). A high percentage (61.24%) of contigs had no protein or nucleotide hits; these may represent novel thrips proteins and non-coding RNA, or they could be derived from the less conserved 3′ or 5′ untranslated regions of genes [36], [49], [50].

### Gene Ontology and KEGG Pathway

**Figure 4:**
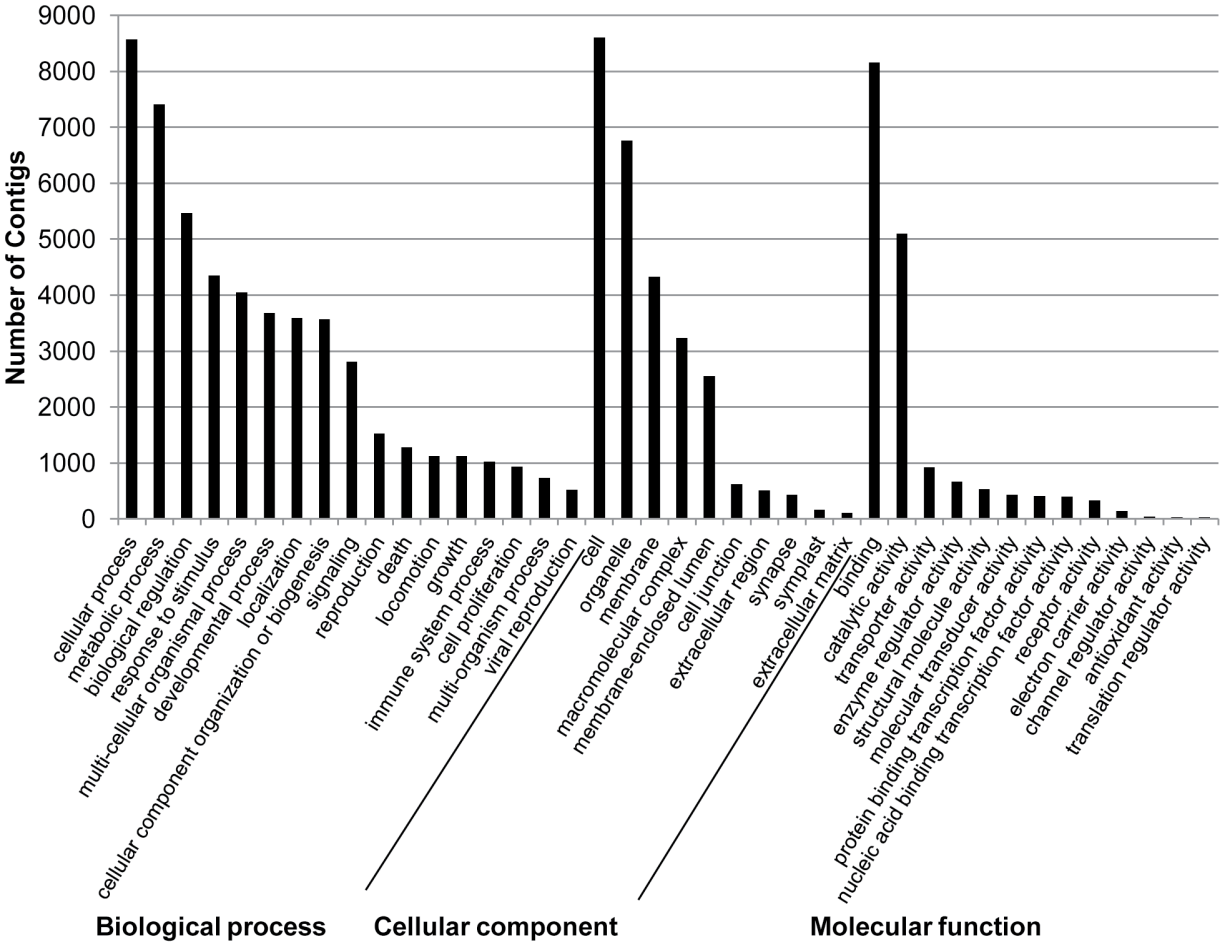
Distribution of *Frankliniella occidentalis* salivary gland contigs by provisional biological processes, cellular component, and molecular function gene ontology (GO) categories. Each category represents a GO term assigned by Blast2GO analysis.

Gene Ontology (GO) terms were assigned to 10,013 contigs (Table S3). The three most common categories in cellular components (Figure 4) were cell, organelle, and membrane. In the molecular function category (Figure 4) the three most common categories were binding, catalytic activity, and transporter activity. In the biological process category (Figure 4) the three most common categories were cellular process, metabolic process, and biological regulation. The most common categories reported herein are consistent with other sialotranscriptomes [35], [36].

Contigs in the sialotranscriptome of *F. occidentalis* were mapped to a total of 129 KEGG pathways (Table S4), the top 20 of which are shown in Table 4. The majority of salivary gland sequences mapped to metabolic pathways including: purine metabolism (346 contigs), pyrimidine metabolism (141 contigs), and inositol phosphate metabolism (79 contigs). Consistent with thrips feeding behaviors there were several enzymes found in predicted KEGG pathways involved in sugar metabolism including: amino sugar and nucleotide sugar metabolism (25 enzymes), starch and sucrose metabolism (24 enzymes), galactose metabolism (18 enzymes), and fructose and mannose metabolism (18 enzymes). Interestingly there were also 17 enzymes found in the porphyrin and chlorophyll metabolism pathway which is consistent with thrips feeding behaviors in which they ingest the full contents of mesophyll cells, including chloroplasts [12], [13].

**Table 4:** Top 20 predicted KEGG pathways in the *Frankliniella occidentalis* sialotranscriptome.

KEGG pathway	Number of contigs	Number of enzymes
Purine metabolism	346	49
Pyrimidine metabolism	141	31
Phosphatidylinositol signaling system	89	19
Glycolysis/gluconeogenesis	81	27
Inositol phosphate metabolism	79	20
Starch and sucrose metabolism	78	24
Amino sugar and nucleotide sugar metabolism	75	25
Glycerophospholipid metabolism	68	21
Glycerolipid metabolism	68	15
Aminoacyl-tRNA biosynthesis	63	23
Pyruvate metabolism	62	24
Oxidative phosphorylation	61	9
Citrate cycle (TCA cycle)	61	19
Lysine degradation	61	13
Thiamine metabolism	61	4
Galactose metabolism	59	18
Arginine and proline metabolism	57	25
Nitrogen metabolism	57	16
Valine, leucine and isoleucine degradation	56	17
Fatty acid metabolism	56	15

### Genes of interest

The *F. occidentalis* sialotranscriptome was screened for several known insect salivary proteins (Table S5). Sequences encoding for metalloprotease (27 contigs), β-glucosidase (16 contigs), short chain acyl-coenzyme A dehydrogenase (11 contigs) and aldehyde dehydrogenase (11 contigs) predominated this search (Table 5). Thrips salivary glands contained several proteins potentially involved in the detoxification and inhibition of plant defenses. Aldehyde dehydrogenases (ALDH's, E.C.1.2.1) catalyze the oxidation of aldehydes into carboxylic acids. ALDH's are ubiquitous across virtually all eukaryotes and as a salivary protein they detoxify aldehydes present in food [51], [52]. The ALDH's (11 contigs) present in the sialotranscriptome of *F. occidentalis* and *Empoasca fabae* (Harris) [36] may play a role in counteracting aldehyde based plant defenses [53]. The other predominant dehydrogenase found in the *F. occidentalis* sialotranscriptome, short chain acyl-coenzyme A dehydrogenase (EC 1.3.1.86) (11 contigs) is involved in the metabolism of short chain fatty acids and has also been found in the sialotranscriptome of the whitefly *Bemisia tabaci* (Gennadius) [35].

**Table 5:** Genes of interest identified in the *Frankliniella occidentalis* sialotranscriptome.

Functional category of genes	Candidate genes	Number of sequences
**Detoxification and inhibition of plant defenses**		
	Metalloprotease*	27
	Aldehyde dehydrogenase	11
	Glucose dehydrogenase	2
	Glucose oxidase	1
	Regulacin	1
**Extra-oral digestion of cell wall components**		
	β-glucosidase	16
	Endo-beta-glucanase	5
	Pectin lyase	2
**Sugar metabolism**		
	Maltase	4
	Sucrase	4
	α-glucosidase	4
	α-amylase	2
**General digestion**		
	Short chain acyl-CoA dehydrogenase	11
	Serine protease	4
	Carboxypeptidase	4
	Chitinase	3
	Lipase	3
	Nucleotidase	1

*This protein may also be associated with metabolism, but it has been hypothesized to be associated with the inhibition of host defenses for a range of insect species.

Glucose oxidase (GOX, EC1.1.3.4) and glucose dehydrogenase (EC1.1.1.47) were also found in the *F. occidentalis* sialotranscriptome. Both of these enzymes are oxidoreductases that have been implicated in the detoxification or inhibition of plant wound responses [1], [40], [54], [55]. Salivary GOX secreted by *Helicoverpa zea* (Boddie) was shown to inhibit wound induced nicotine production in *Nicotiana tabacum* L. [1], [56] and the presence of GOX in *Myzus persicae* (Sulzer) saliva is hypothesized to explain the weak induction of wound responses in aphid infested plants [40]. Glucose dehydrogenase has been identified from several aphid species and is implicated in the detoxification of plant defensive compounds [37], [39], [40], [55], [57]. We hypothesize that the GOX (1 contig) and glucose dehydrogenases (2 contigs) found in the *F. occidentalis* sialotranscriptome may play a role in the counteraction of mesophyll based plant defenses during thrips feeding. Interestingly we also found a regucalcin (1 contig), which is a Ca^2+^ binding protein found in the saliva of *A. pisum* and *B. tabaci* [35] that has been implicated in inhibiting calcium mediated plugging of sieve elements [39]. We hypothesize that the regucalcin found in the *F. occidentalis* sialotranscriptome may play a role in inhibiting calcium mediated defenses in mesophyll cells, or may simply play a role in the regulation of calcium dependent intracellular signaling.

Several proteases were found in the salivary glands. Insect digestive proteases play two essential roles in an insects physiology, they inactivate protein toxins or proteins used in defense responses, and they break down proteins into free amino acids making them nutritionally available. Metalloproteases play a major role in biological processes of many different organisms [58]. Metalloproteases have been identified as major protein components of *A. pisum* and *I. scapularis* saliva [39], [59] and are also essential for successful feeding by *I. ricinus* (L.) [60]. The metalloproteases in tick saliva and snake venom are hypothesized to counteract host defenses by breaking down proteins essential to the coagulation response [59], [61]. In aphids, metalloproteases are also hypothesized to counteract host defenses, by degrading phloem defense response proteins [39]. In thrips, these abundant proteases may play a role in degrading proteins associated with host defenses that occur in mesophyll tissues. While metalloproteases may be involved in degrading proteins for amino acid release [39], serine proteases are the most common salivary protease attributed to digestion of dietary proteins by phytophagous insects [62]–[64]. Interestingly, serine proteases have also been implicated in the digestion of insoluble structural proteins of insect prey [65] and in the digestion of proteins from pollen grains [66]. *F. occidentalis* is an omnivore [67] and the serine proteases (4 contigs) found in the salivary glands may contribute to protein digestion from plant tissue, mite eggs, or pollen grains, and the chitinases (3 contigs) we identified may play a role in the digestion of fungal spores.

On leaves, in addition to sucking out the contents of individual cells, thrips may engage in prolonged ingestion, likely from areas where cells have been damaged by thrips stylets and extra-orally digested by thrips salivary enzymes [12]–[15]. Large areas of damage are visible within leaves after prolonged exposure to *F. occidentalis* feeding, in which pockets of cells are missing and only remnants of cell walls and organelles remain [15]. We found several enzymes in the *F. occidentalis* sialotranscriptome that are involved in extra oral digestion of cell wall components and cell contents (Table 5). The major polysaccharide component of plant cell walls is cellulose, a linear polymer of β-1,4-linked D-glucose units. Cellulase is a general term used to describe cellulytic enzymes that catalyze the hydrolysis of the β-1,4-glycosidic bonds in cellulose. These enzymes allow phytophagous insects to penetrate their stylets through the intercellular matrix and breakdown plant cell walls to release cell contents for subsequent ingestion. β-glucosidases (EC 3.2.1.21), which hydrolyze cellobiose to glucose from non-reducing ends, are common in most insects [68] and have been reported in the saliva and salivary glands of *A. pisum* and *E. fabae* [36], [40]. Endo-β-1,4-glucanase (EC 3.2.1.4), is another cellulase which randomly cleaves amorphous sites of cellulose chains. These cellulases have been reported from a limited number of insects compared to β-glucosidases [68]; however, endo-β-1,4-glucanase has been found in the saliva or salivary glands of a diverse number of insects including: the hemipterans *Schizaphis graminum* (Rondani) [54], [69], *A. pisum* [68], *Homalodisca vitripennis* (Germar) [70], and *E. fabae* [36], the termite *Reticulitermes speratus* (Kolbe) [71], and the lepidopteran *Helicoverpa armigera* (Hübner) [50]. The presence of both β-glucosidases (16 contigs) and endo-β-1,4-glucanases (5 contigs) is consistent with the feeding damage inflicted by *F. occidentalis*.

Pectin is another major polysaccharide component in plant cell walls and the middle lamella. Phytophagous insects produce various types of hydrolytic enzymes that degrade pectin, generically referred to as pecitnases. The secretion of salivary pectinases may induce subtle degenerative changes in plant cells making them more accessible to probing behaviors [54], [72] or they may cause the release of nutrients from large areas of degraded tissue [6]. Polygalacturonases, pectinases that specifically hydrolize unsubstituted α-1,4-polygalacturonic acid linkages, were shown to be the main cause of Lygus bug feeding damage [5], [6]. Salivary pectinases have been found in the saliva and salivary glands of many different hemipterans [5], [6], [36], [54], [72], [73] and the presence of pectin lyase (EC 4.2.2.2) in the *F. occidentalis* sialotranscriptome is also consistent with the types of feeding damage it inflicts.

In addition to breaking down cell wall components, thrips saliva is also likely involved in the metabolization of complex sugars. We found several sugar degrading enzymes including α-amylase (EC 3.2.1.1) (2 contigs) [35], [36], [40], [49], [50], [57], [74], [75], α-glucosidase (EC 3.2.1.20) (4 contigs) [40], [66], [76], [77], maltase (EC 3.2.1.20) (4 contigs) [30], [78], and sucrase (EC 3.2.1.48) (4 contigs) [35], that are commonly found in the salivary and hypopharyngeal glands of insects that feed on plant cell contents and nectar. α-amylase breaks down oligosaccharides and polysaccharides by catalyzing the hydrolysis of α-1,4-D glucosidic linkages. Starch is a common polysaccharide found in plants and is metabolized by a complex of α-amylases including α-glucosidase and maltase [36], [57], [75]. The presence of sugar degrading enzymes in the salivary glands of *F. occidentalis* is consistent with the secretion of thrips saliva for extra-oral digestion.

## Conclusions

This is the first sialotranscriptome study of any Thysanopteran. The minute size of thrips is a limiting factor for studying individual body parts and tissues; however, here we have shown that it is possible to obtain high quality sequences of a gland that is comprised of only 8 cells. This study was performed in an effort to better understand how thrips feed and to create a tool with which to study how Tospovirus infection of the thrips vector affects components of thrips saliva. The enrichment analysis showed that metabolism is a main function of thrips salivary glands. Furthermore we found that although thrips feeding behaviors are very different from those of aphids and whiteflies, thrips salivary proteins are similar to those reported for Hemipterans. Based on the identified thrips salivary proteins, it appears that thrips saliva plays a role in extra-oral digestion of plant tissues and sugars. Interestingly we also found genes implicated in counteracting plant defense responses. This study has provided a valuable resource to further our understanding of thrips interactions with tospoviruses and their host plants.

## Supporting Information

Table S1 ***Frankliniella occidentalis*** **sialotranscriptome contig annotation.** The following information is provided for each contig: salivary gland assembly contig ID, InterproScan results, number of GO terms assigned to the protein blast hits by Blast2GO, number of protein blast hits, lowest e-value of any protein blast hit, mean similarity of protein blast hits, protein sequence description assigned by Blast2GO, number of nucleotide blast hits, lowest e-value of any nucleotide blast hit, mean similarity of nucleotide blast hits, nucleotide sequence description assigned by Blast2GO, nucleotide inference, lowest E-value (protein or nucleotide), and highest similarity to protein or nucleotide.
(XLSX)

Table S2 **Descriptive information on the 12,166** ***Frankliniella occidentalis*** **sialotranscriptome contigs with significant BLAST hits (E-value≤1.0E^−6^), includes: information on alignment and hit description.**
(XLSX)

Table S3 **Descriptive information on gene ontology (GO) for** ***Frankliniella occidentalis*** **sialotranscriptome contigs, includes: blast hit description, GO group, GO-ID, and GO term.**
(XLSX)

Table S4 **Descriptive information on the KEGG pathway analysis for the** ***Frankliniella occidentalis*** **sialotranscriptome, includes: pathway descriptions, number of sequences in each pathway, enzyme names, enzyme ID and number of enzyme sequences.**
(XLSX)

Table S5 **Screening of the** ***Frankliniella occidentalis*** **sialotranscriptome known insect salivary proteins, information includes: the name of the reference gene used to search the sialotranscriptome, protein name,** ***F. occidentalis*** **sialotranscriptome contig name that matched the reference gene, (E-value≤1.0E^−6^) and the E-value.**
(XLSX)
